# Transporter-Mediated Delivery of Small Molecule Drugs to the Brain: A Critical Mechanism That Can Advance Therapeutic Development for Ischemic Stroke

**DOI:** 10.3390/pharmaceutics12020154

**Published:** 2020-02-14

**Authors:** Erica I. Williams, Robert D. Betterton, Thomas P. Davis, Patrick T. Ronaldson

**Affiliations:** Department of Pharmacology, College of Medicine, University of Arizona, 1501 N. Campbell Avenue, P.O. Box 245050, Tucson, AZ 85724-5050, USA; eiwilliams@email.arizona.edu (E.I.W.); rdbetter@email.arizona.edu (R.D.B.); davistp@email.arizona.edu (T.P.D.)

**Keywords:** blood-brain barrier, drug delivery, HMG-CoA reductase inhibitors, organic anion transporting polypeptides, neurovascular unit, stroke, transporter

## Abstract

Ischemic stroke is the 5th leading cause of death in the United States. Despite significant improvements in reperfusion therapies, stroke patients still suffer from debilitating neurocognitive deficits. This indicates an essential need to develop novel stroke treatment paradigms. Endogenous uptake transporters expressed at the blood-brain barrier (BBB) provide an excellent opportunity to advance stroke therapy via optimization of small molecule neuroprotective drug delivery to the brain. Examples of such uptake transporters include organic anion transporting polypeptides (OATPs in humans; Oatps in rodents) and organic cation transporters (OCTs in humans; Octs in rodents). Of particular note, small molecule drugs that have neuroprotective properties are known substrates for these transporters and include 3-hydroxy-3-methylglutaryl coenzyme A (HMG-CoA) reductase inhibitors (i.e., statins) for OATPs/Oatps and 1-amino-3,5-dimethyladamantane (i.e., memantine) for OCTs/Octs. Here, we review current knowledge on specific BBB transporters that can be targeted for improvement of ischemic stroke treatment and provide state-of-the-art perspectives on the rationale for considering BBB transport properties during discovery/development of stroke therapeutics.

## 1. Introduction

Stroke epidemiology in the United States is characterized by 795,000 new incidences each year [1]. Ischemic strokes comprise approximately 87% of all strokes, which is in contrast to hemorrhagic strokes that are much less common [1]. Ischemic stroke pathophysiology involves reduced delivery of oxygen and glucose to an affected brain region due to impaired blood supply, a process that leads to an irreversibly damaged infarct core as well as potentially salvageable surrounding tissue known as the penumbra [2]. Treatment of the ischemic core is virtually impossible due to rapid development of necrosis; however, the penumbra is a primary target for therapeutic intervention due to slower cell degradation [2,3,4]. To date, recombinant tissue plasminogen activator (r-tPA; Activase^®^ (alteplase)) is the only drug approved for clinical use as a stroke therapeutic in the United States. Therapy with r-tPA is limited by its narrow therapeutic window (i.e., 4.5 h) and/or risk of intracerebral bleeding complications [3]. More recently, reperfusion therapy via mechanical endovascular thrombectomy (EVT) has provided considerable benefits to stroke patients including a marked improvement in reperfusion [5,6]; however, many patients who receive EVT remain severely disabled [6,7,8,9]. As noted by the National Institute for Neurological Disorders and Stroke (NINDS), stroke treatment paradigms include therapy immediately after the stroke as well as preventative measures and post-stroke rehabilitation (Table 1). Therapy with r-tPA and EVT are the only two measures that are currently available as immediate therapeutic strategies to treat ischemic stroke. Both r-tPA and EVT involve recanalization (i.e., reperfusion) of ischemic brain tissue, a process that can exacerbate neuronal injury. Central nervous system (CNS) damage following recanalization ranges in severity from ischemic core enlargement to development of edema or fatal hemorrhaging, a critical component of ischemia/reperfusion (I/R) injury [10,11]. Pathophysiological processes associated with I/R injury include enhanced cerebrovascular permeability and leakage, activation of cell death mechanisms (i.e., apoptosis, autophagy, necrosis), and increased production of reactive oxygen species (i.e., oxidative stress) [11,12,13].

**Figure 1:**
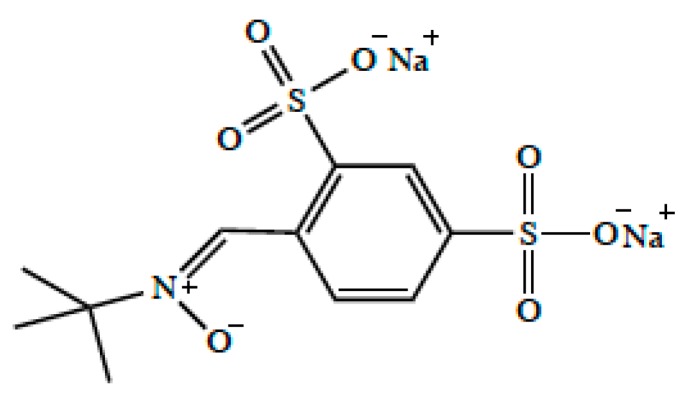
Chemical structure of disufenton sodium (i.e., NXY-059, Cerovive^®^).

The frequent use of reperfusion therapies in stroke treatment highlights an unmet clinical need for strategies that can both protect neuronal tissue from further ischemic damage or promote neuronal repair following I/R injury. Indeed, a vast array of currently marketed drugs and experimental therapeutics have been reported to possess neuroprotective properties in preclinical stroke studies. In fact, 95% of published studies between 1990 and 2018 reported positive neuroprotective outcomes in animal stroke models; however, none of these studies have been successfully translated to Phase III clinical trials [6]. There are several reasons for mismatches between preclinical successes in neuroprotection and failed clinical trials including (i) treatments in preclinical studies largely focus on measurements of infarct volumes and not on functional neurocognitive outcomes; (ii) use of large preclinical doses in animal models that cannot be safely extrapolated to human populations; and (iii) preclinical models are primarily conducted in young animals while stroke is highly prevalent in elderly men and women [6,14]. An additional consideration is that studies examining neuroprotection in experimental stroke have not evaluated specific biological mechanisms required for drugs to efficiently permeate the blood-brain barrier (BBB). A pointed example of the need to consider BBB permeability in stroke drug development is the preclinical and clinical advancement of disufenton sodium (i.e., NXY-059, Cerovive^®^; Figure 1), an antioxidant drug. Preclinical studies with disufenton sodium in rodents [15,16] and primates [17] showed that this compound could reduce infarction volumes and improve functional deficits in the setting of experimental stroke. In vitro studies in cultured brain endothelial cells showed that disufenton sodium could also protect against increased paracellular permeability induced by oxygen/glucose deprivation conditions [18]. Although effective and safe plasma concentrations could be achieved in humans [19,20], this compound ultimately failed in clinical trials. One of the primary reasons for this failure is that disufenton sodium is a polar molecule with limited hydrophobicity, a property that greatly reduced its BBB permeability and ability to achieve effective concentrations in the ischemic brain. Additionally, disufenton sodium is known to be a substrate for organic anion transporters (OATs in humans; Oats in rodents) in the kidney [21]; however OATs/Oats at the BBB are not involved in blood-to-brain drug transport, a fact that further limits the ability of disufenton sodium to reach efficacious concentrations in the ischemic brain.

**Figure 2:**
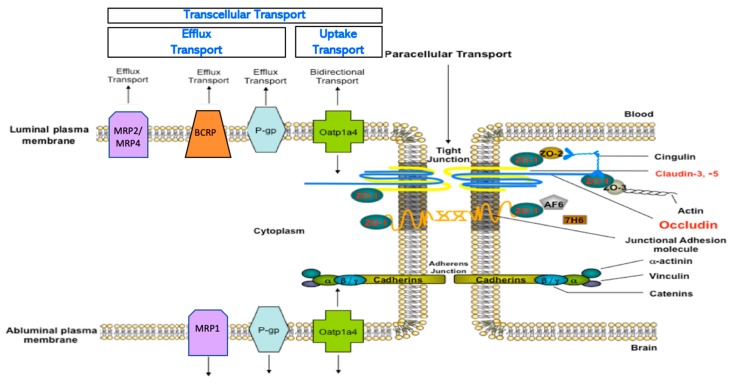
Physical and biochemical characteristics of the blood-brain barrier (BBB). Physiologically, paracellular diffusion is restricted by tight junction protein complexes and adherens junctions. Transporters contribute to biochemical barrier properties. Specifically, efflux transporters such as P-glycoprotein (P-gp), Breast Cancer Resistance Protein (BCRP), and Multidrug Resistance Proteins (MRPs) limit the ability of drugs to permeate the BBB and accumulate in brain parenchyma. The BBB also possesses uptake transporters such as organic anion transporters in humans/rodents (OATPs/Oatps), which can facilitate selective uptake of therapeutics into the brain. While organic cation transporters (OCTs) have also been detected at the BBB, their exact localization in brain microvascular endothelial cells is unknown.

The experience with disufenton sodium emphasizes that discovery of new drugs and/or development of novel approaches to treat ischemic stroke require detailed evaluation of specific mechanisms that can facilitate successful CNS drug delivery. By extension, such an endeavor requires a thorough understanding of physical and biochemical properties of the BBB with an emphasis on how the BBB can be targeted to ensure attainment of efficacious drug concentrations in the brain. At the biochemical level, the BBB possesses numerous endogenous transport proteins that allow for selective uptake of required nutrients or restrict brain accumulation of potentially toxic substances (Figure 2) [22,23,24]. Published studies on transporters in stroke have primarily focused on pathophysiological implications of altered BBB transport of ions [25,26,27] and glucose [28,29]. With respect to drugs, there are a few reports on changes in brain-to-blood drug transport by efflux transporters following focal cerebral ischemia [30,31,32] and no publications on transporters that can directly deliver neuroprotective drugs to the brain for stroke pharmacotherapy. This is a significant knowledge gap that must be addressed by new experimental evidence. In this review, we highlight key features of the BBB with an emphasis on endogenous BBB transporters that have considerable potential to be targeted for optimization of CNS delivery of small molecule therapeutics. This objective is critical to the discovery and/or development of new chemical entities that can improve neurocognitive outcomes following ischemic stroke. 

## 2. The Blood-Brain Barrier and the Neurovascular Unit

It is generally accepted that the BBB exists at the level of the brain microvascular endothelium. A central concept in BBB physiology is that endothelial cells are not intrinsically capable of forming a “barrier” between cerebral tissue and the systemic circulation. Rather, barrier characteristics can only be acquired through coordinated intercellular communication [13,33]. This relationship implies the existence of a “neurovascular unit (NVU)”, a concept formally defined in 2001 at the Stroke Progress Review Group meeting of the National Institute of Neurological Disorders and Stroke (NINDS) of the National Institutes of Health (NIH) [34]. By emphasizing the symbiotic nature of cell-cell interactions between endothelial cells, glial cells (i.e., astrocytes, microglia), pericytes and neurons as well as contributions from constituents of the extracellular matrix, the concept of the NVU caused an ideological shift in our understanding of neurological diseases [34]. Instead of acting as independent entities, it is now understood that cellular compartments of the CNS interact directly with the cerebral microvasculature to enable endothelial cells to develop distinct barrier properties and to allow for dynamic BBB responses to pathological stressors. In fact, BBB injury is an early pathological event in ischemic stroke that occurs prior to onset of neuronal damage [35,36]. The pathophysiological mechanisms of BBB dysfunction in ischemic stroke is beyond the scope of this review. For details on mechanisms involved in the BBB response to stroke, the reader is directed to a recent publication by our group [13].

### 2.1. Endothelial Cell Junctions

Physiological functioning of the CNS requires precise regulation of the extracellular space in brain parenchyma. Therefore, the BBB must possess mechanisms that can facilitate nutrient transport, precisely regulate ion balance, and prevent CNS uptake of potentially toxic substances. Despite only accounting for 2% of total body mass, the adult human brain consumes approximately 20% of total body energy requirements [37]. Such requirements imply a need for specific physical and biochemical characteristics of cerebral endothelial cells that generate BBB properties. BBB endothelial cells possess unique anatomical features such as lack of fenestrations, a paucity of pinocytotic activity, and presence of tight junction/adherens junction protein complexes [13]. Tight junction protein complexes form the physical “seal” of the BBB and limit paracellular diffusion [13,38]. Tight junctions are prominently involved in the regulation of water and solute movement between endothelial cells (i.e., paracellular diffusion) via formation of a continuous and impermeable barrier. Transmembrane tight junction proteins include claudins, occludin, junctional adhesion molecules (JAMs), and tricellulin. Claudins, in particular claudin-5, are central to formation of the physiological barrier of the brain microvasculature and thereby restrict small molecule uptake into the brain via paracellular diffusion [39,40]. Occludin is also a critical regulator of BBB functional integrity, predominantly via its ability to assemble into dimers and higher-order oligomers [41,42,43]. Loss of JAM protein expression and/or trafficking of JAM proteins away from the endothelial cell plasma membrane leads to a loss of BBB properties [44,45]. Similarly, reduced expression of tricellulin in brain microvascular endothelial cells is associated with increased BBB permeability [46]. Transmembrane tight junction proteins are directly linked to the cytoskeleton by interactions with accessory proteins known as zonula occluden (ZO) proteins [13]. ZO proteins are members of the membrane-associated guanylate kinase-like (MAGUK) protein family. Several reports in the scientific literature have shown that dissociation of ZO-1 from the actin cytoskeleton is associated with increased BBB permeability [47,48,49], suggesting that ZO-1 is critical to stability and function of the tight junction. Other MAGUK isoforms (i.e., ZO-2, ZO-3) have been detected at the tight junction; however, the exact role of these proteins in BBB integrity is poorly understood [13].

Paracellular permeability at the BBB is also regulated by adherens junctions, which are specialized cell-cell interactions formed by cadherins and associated proteins that are directly linked to actin cytoskeletal filaments [50]. Cadherins activate phosphoinositide 3-kinase signaling, an essential mechanism for maintenance of endothelial integrity and for spatial organization during angiogenesis [51]. Brain microvascular endothelial cells express high levels of cadherin-10 relative to VE-cadherin [52]. In contrast, CNS vascular structures that are devoid of BBB properties such as circumventricular organs and choroid plexus capillaries primarily express VE-cadherin [52]. Cadherin proteins directly associate with catenins, a property that is required to ensure optimal cadherin function. At least four catenin isoforms (i.e., α, β, χ, and p120) are detectable in brain microvascular endothelial cells. Perhaps the most critical interaction involves β-catenin, which binds to cadherin and α-catenin, thereby linking this protein complex to the actin cytoskeleton [53]. β-catenin also interacts directly with the Wnt signaling pathway at the brain microvascular endothelium, an effect that contributes to development of the BBB phenotype [54,55]. An additional protein constituent of adherens junctions is agrin, a heparin sulfate proteoglycan that promotes cadherin and catenin localization to endothelial cell junctions [56]. Under pathological conditions, expression of cadherins and catenins at the paracellular cleft between adjacent endothelial cells is associated with induction of BBB repair mechanisms [57].

### 2.2. Endogenous BBB Transporters

Transporters are a critical biochemical mechanism at the BBB that permit brain entry of some small molecules while restricting brain permeability of others [4,23,24]. Such transporters include ATP-binding cassette (ABC) transporters and solute carrier (SLC) transporters. ABC transporters hydrolyze ATP to generate the biological energy required to transport drugs and metabolites against their concentration gradient. Therefore, these transporters constitute a formidable challenge in the treatment of neurological diseases by greatly restricting drug delivery into brain tissue from the systemic circulation. Cerebral endothelial cells have increased content of mitochondria, which enables generation of biological energy required to drive solute transport across the microvascular endothelium [58]. ABC transporters that are localized to the brain microvascular endothelium include P-glycoprotein (P-gp), breast cancer resistance protein (BCRP in humans; Bcrp in rodents), and Multidrug Resistance Proteins (MRPs in humans; Mrps in rodents). Perhaps the most critical efflux transporter that is expressed at the BBB is P-gp, which is known to restrict brain permeability of hundreds of structurally diverse xenobiotics (molecular weight range 100–4000 Da) [59]. It is noteworthy that many P-gp substrates are also transported by BCRP/Bcrp. In fact, BCRP/Bcrp is known to function synergistically with P-gp to limit CNS drug permeation [60]. Therefore, it is critical that both transporters be considered in assessing drug transport properties during preclinical development. In contrast to P-gp and BCRP/Bcrp, MRPs/Mrps are known to efflux anionic drugs and their glucuronidated, sulfated, and glutathione-conjugated metabolites [23,61,62]. Due to their role in limiting drug accumulation in the CNS, efflux transporters have long been the focus of considerable research in neuropharmacology. The goal of such studies was to demonstrate that pharmacological inhibition of efflux transporters could result in improved CNS disposition of therapeutic compounds. Indeed, enhanced CNS uptake of drugs resulting from pharmacological inhibition or genetic knockout of efflux transporters has been demonstrated in many animal models [63,64,65,66,67]; however, such significant increases in brain penetration of efflux transporter substrates is unlikely in the clinical setting [68]. Furthermore, clinical utility of selective inhibitors for efflux transporters are limited by toxicity concerns and unpredictable pharmacokinetics [24]. For example, the second generation P-gp inhibitor valspodar (PSC833) is known to inhibit hepatic metabolism of paclitaxel, an effect that can lead to unsafe plasma concentrations of paclitaxel [69]. It is possible that targeting intracellular signaling pathways can provide an approach to control P-gp mediated drug efflux for optimization of CNS drug delivery while avoiding the negative effects of direct pharmacological inhibition. For example, targeting sphingolipid signaling with fingolimod reduced basal P-gp activity and improved CNS drug delivery [70,71]. It is important to point out that suppression of basal P-gp function may render the brain susceptible to toxic xenobiotics, a fact that would greatly limit the utility of this approach for improvement of CNS drug delivery. 

**Figure 3:**
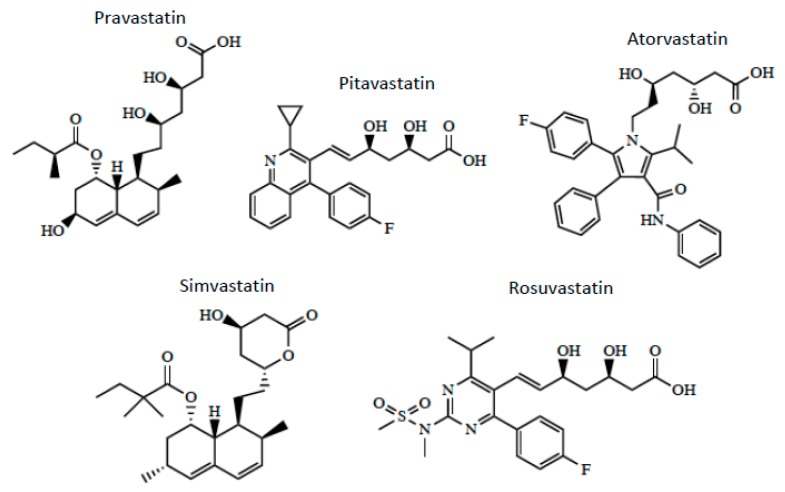
Chemical structures of HMG-CoA reductase inhibitors (i.e., Statins).

**Figure 4:**
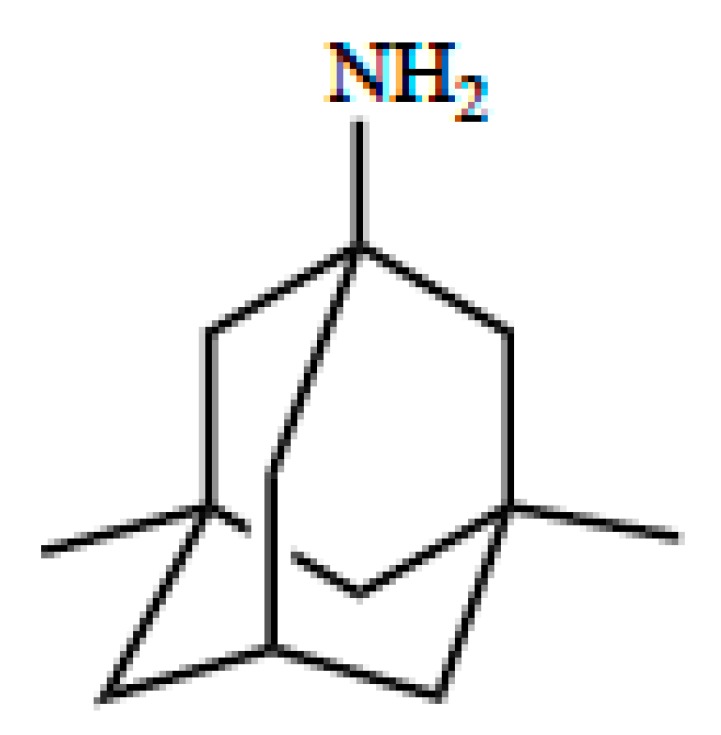
Chemical structure of memantine.

Optimization of CNS drug delivery for treatment of neurological diseases such as ischemic stroke can be better accomplished through identification and characterization of endogenous BBB uptake transporters. Indeed, blood-to-brain transport of circulating solutes involves members of the solute carrier (SLC) superfamily of transporters. In humans, there are 395 membrane-spanning SLC transporters that have been identified, which are organized into 52 families [72]. Transport mechanisms for SLC transporters include facilitated diffusion (i.e., transport in the direction of the substrate concentration gradient) or secondary/tertiary active transport (i.e., substrate transport driven by gradients established by other primary or secondary active transporters) [4]. Regardless of the nature of transport, SLC members exhibit different specificities and affinities for various structurally diverse substrates including therapeutic agents [4,23,73]. SLC transporters that can facilitate blood-to-brain delivery of drugs relevant to ischemic stroke treatment include organic anion transporting polypeptides (OATPs in humans; Oatps in rodents) and organic cation transporters (OCTs in humans; Octs in rodents). As shown in Table 2, OATPs/Oatps transporter substrates tend to be amphipathic nature [24,74]. For example, OATPs/Oatps transport 3-hydroxy-3-methylglutaryl coenzyme A (HMG-CoA) reductase inhibitors (i.e., statins; Figure 3), which have been shown to exhibit both neuroprotective and antioxidant properties [75,76,77]. The primary Oatp isoform that is involved in blood-to-brain drug transport at the human BBB is OATP1A2 [77,78]. In situ hybridization histochemistry, immunofluorescence microscopy, and western blot analysis of isolated cerebral microvessels have confirmed localization and/or expression of Oatp1a4 (i.e., the rodent orthologue of OATP1A2) at the rodent BBB [74,76,79,80]. Additionally, there is evidence for expression of organic cation transporters (OCTs) at the BBB [81,82]. Specifically, OCT1, OCT2, and OCT3 are expressed at the human brain microvascular endothelium; however, the exact localization of these transporters remains controversial [81]. There is some in vitro data from rat brain microvessel endothelial cells that has demonstrated Oct1/rOct2 expression with localization primarily at the luminal plasma membrane [83]. It is generally agreed that expression of OCT/Oct isoforms at the BBB is relatively high, a fact that points towards a role for these transporters in the brain uptake of cationic drugs [81]. In fact, several cationic compounds may be efficacious as stroke therapeutics. For example, 1-amino-3,5-dimethyladamantane (i.e., memantine; Figure 4), an *N*-methyl-d-aspartate (NMDA) receptor antagonist that has been previously used in treatment paradigms for ischemic stroke, is an established transport substrate for OCT1/OCT2 [83]. The exact mechanism of memantine transport across the BBB requires more detailed investigation; however, therapeutic targeting of memantine to the CNS via OCT-dependent drug delivery may prove to be an effective mechanism to enhance the utility of this neuroprotective drug in treatment of ischemic stroke. Other OCT transport substrates that may be effective as stroke therapeutics include the D2/D3 receptor agonist pramipexole [84,85], the monoamine oxidase inhibitor selegiline [86], and the nicotinic receptor agonist varenicline [87]. Further data is required to determine the functional role of OCT isoforms expressed at the BBB in the setting of ischemic stroke.

## 3. OATP/Oatp-Mediated Transport of Statins

Both OATP1A2 and Oatp1a4 are sodium-independent transporters that rely on the transmembrane concentration gradient to facilitate movement of drugs into the brain. Evidence for Oatp-mediated uptake of statins into brain tissue has been obtained from studies in Oatp1a4 knockout mice that showed reduced blood-to-brain transport of various statin drugs as compared with age-matched wild-type control mice [88]. Our laboratory has demonstrated that enhanced CNS delivery of atorvastatin via Oatp1a4 following hypoxic stress provides neuroprotective effects as indicated by attenuation of poly-ADP ribose polymerase (PARP) cleavage [75]. An increase in CNS expression of cleaved PARP protein is an established biomarker of neuronal apoptosis. We have also shown that atorvastatin uptake is significantly greater in female Sprague-Dawley rats as compared to age-matched male rats [88]. This effect is directly related to a 4-fold higher BBB expression of Oatp1a4 in females as compared to males. Interestingly, castration of male rats resulted in Oatp1a4 protein expression and atorvastatin brain uptake levels that were not different from age-matched females, an effect that may suggest that male sex hormones repress Oatp1a4 functional expression at the BBB [88]. 

**Figure 5:**
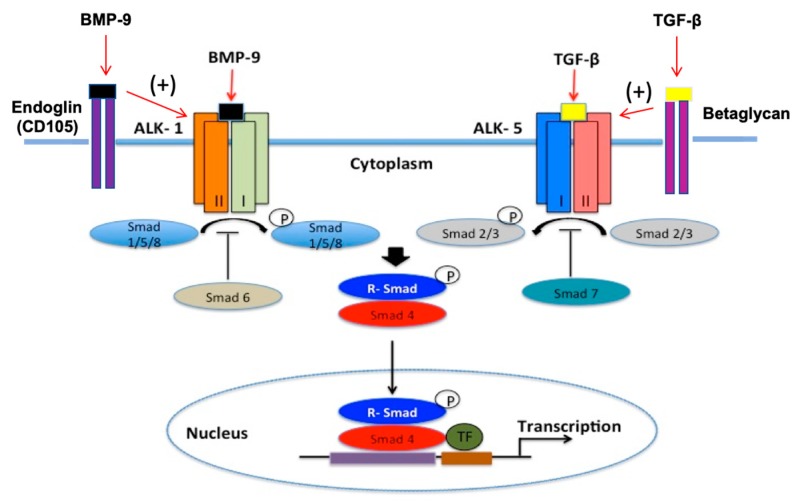
Transforming growth factor-β (TGF-β) signaling at the brain microvascular endothelium.

To optimize statin delivery across the BBB, functional expression of OATP/Oatp must be tightly controlled. This can be achieved by targeting transforming growth factor β (TGF-β) signaling. Briefly, TGF-β cytokines bind type I serine/threonine kinase receptors (i.e., activin receptor-like kinase (ALKs)) and recruit type II receptors to form a heterotetrameric complex (Figure 5). At the BBB, intracellular signals via the TGF-β pathway are propagated by ALK1 and ALK5 through phosphorylation of small signal transduction proteins known as Smads. Phosphorylated Smad proteins are then able to bind with the common Smad (i.e., Smad4), which enables translocation of this protein complex to the nucleus and activation of transcription of target genes [4,24]. Additionally, ALK5 receptor expression is required for TGF-β/ALK1 activation whereas the ALK1 pathway inhibits TGF-β/ALK5-Smad signaling [89]. Type III TGF-β receptors (i.e., endoglin, betaglycan) present another mechanism for regulation of TGF-β pathway specificity. That is, efficient TGF-β/ALK1 signaling requires endoglin (CD105) activity whereas betaglycan performs a similar role for the TGF-β/ALK5 pathway [90,91,92,93]. ALK1 and ALK5 receptors act in an opposing manner at the BBB [90,93,94,95,96,97]. In accordance with this known physiology, we have shown opposite effects of these signaling pathways on Oatp1a4 expression and activity [76,79,80,98]. Specifically, activation of TGF-β/ALK1 signaling increases Oatp1a4 protein expression and uptake transport for atorvastatin and pravastatin at the BBB [80,98]. Similarly, inhibition of the TGF-β/ALK5 pathway enhances Oatp1a4 functional expression at the microvascular endothelium, an effect that leads to improved blood-to-brain transport of atorvastatin [75]. These observations provide mechanistic proof that targeting TGF-β signaling during ischemic stroke is an approach that can control brain uptake of Oatp substrate drugs such as statins.

## 4. Neuroprotective Properties of Statins in Stroke 

Statins have long been used for the management of patients with high serum concentrations of low-density lipoprotein (LDL) cholesterol. This class of drugs targets HMG-CoA reductase to inhibit the conversion of HMG-CoA to mevalonate in the liver. Independent of their well-documented effects as cholesterol-lowering drugs, there is increasing evidence that statins exhibit neuroprotective properties. Indeed, such effects have been observed in clinical practice. It is well known that administration of statins prior to onset of ischemic stroke is associated with a lower degree of physical disability [99,100,101]. Statins also improve outcomes in patients after stroke onset. For example, atorvastatin is associated with reduced risk of recurrent stroke in patients with a recent stroke or transient ischemic attack [102,103]. In a study of 104 patients receiving intravenous (i.v.) tissue-type plasminogen activator, post-stroke neurological recovery was improved in patients receiving simvastatin [104]. A randomized controlled study in hemispheric stroke patients showed that statin withdrawal resulted in a 4.6-fold increase in mortality and an 8.7-fold increase in risk of early neurological deterioration [105]. Additionally, preclinical studies have shown that 10 day rosuvastatin treatment reduced infarction volume in a dose-dependent manner in 129/SV WT mice subjected to middle cerebral artery occlusion (MCAO) [106]. Indeed, statins are one of the few drug classes that have shown efficacy in Phase II/III trials of stroke patients and improved neurological outcomes in preclinical stroke studies. Statins can provide neuroprotection through modulation of multiple physiological mechanisms including (i) reduction of inflammation [107,108]; (ii) attenuation of oxidative stress [109,110]; (iii) inhibition of matrix metalloproteinase 9 (MMP-9) activity [110,111,112]; and (iv) regulation of nitric oxide synthase activity [112,113]. For details on statin effects at these specific molecular targets, the reader is directed to a recent review by our group [4].

In addition to their ability to improve post-stroke functional outcomes, statins are safe drugs where therapeutic benefits outweigh risks [82,113,114,115]. Even at high doses, fewer than 1% of patients experience hepatotoxicity as indicated by increased plasma transaminase levels [115]. Another adverse event that is commonly linked to statin administration is rhabdomyolysis. In a randomized controlled trial where patients received 10–80 mg/day, rhabdomyolysis occurred in only 0.1% of statin-treated subjects compared with 0.04% of patients in the placebo group [113]. There have been reports of CNS adverse effects associated with statins such as cognitive impairment and memory loss [82]. Indeed, the FDA identified 60 patients with statin-associated memory loss during a study conducted between 1997 and 2002. These patients received either atorvastatin, pravastatin, or simvastatin and adverse effects were observed within 2 months of treatment; however, all neurological symptoms resolved following discontinuation of statin therapy [113]. Clearly, occurrence of these side effects in patients receiving statins do not preclude administration of these drugs to reduce CNS injury and improve clinical outcomes in stroke patients.

## 5. OCT/Oct-Mediated Transport of Memantine

Although other OCT transport substrates have shown beneficial effects in preclinical stroke models, this review will focus on OCT-mediated transport of memantine because this is the only known OCT substrate drug to have been used clinically in stroke patients. Memantine is a small molecule drug with a pK_a_ of 10.27. At physiological pH (i.e., 7.4), the ratio of positively charged memantine to uncharged memantine is 740.74 as calculated using the Henderson-Hasselbalch equation. While uncharged memantine can cross biological membranes by passive diffusion, the large proportion of charged memantine molecules indicates the need for a transport mechanism to enable this neurotherapeutic to achieve effective concentrations in brain tissue. To date, transport properties of memantine at the BBB have not been fully elucidated; however, memantine has been demonstrated to be a substrate for OCT1/Oct1 and OCT2/Oct2. OCTs/Octs are members of the SLC22A family of transporters and function as electrogenic, facilitative transporters where transport is dependent upon the electrochemical gradient of the organic cation substrate [82]. In mammalian cells, the inside-negative membrane potential is used to drive uptake of organic cations [82]. Confocal microscopy analysis and transmission electron microscopy/immunogold cytochemistry of human and/or rat brain microvascular endothelial cells (BMECs) has confirmed expression of OCT1/Oct1 and OCT2/Oct2 in these cells [82,83]. Additionally, western blot experiments on isolated luminal and abluminal membrane fractions of BMECs showed predominantly luminal expression for both OCT1/Oct1 and OCT2/Oct2 [83]. A recent study demonstrated that memantine uptake via in situ transcardiac perfusion in Swiss outbred mice was dependent upon OCT/Oct mediated transport [116]. More recently, cellular memantine uptake was shown to be increased in the presence of an enhanced outwardly-directed proton gradient, an effect that indicates that the organic cation/carnitine transporter (OCTN) 1 may be involved in blood-to-brain delivery of this neurotherapeutic [117,118]. In contrast, studies in the human brain endothelial cell line hCMEC/d3 reported that memantine uptake was not inhibited by ergothioneine, an OCTN1 transport substrate [118]. At present, the exact mechanism of memantine transport across the BBB requires more extensive investigation. However, therapeutic targeting of memantine to the brain via OCT/Oct-dependent drug delivery may prove to be an effective strategy to optimize the utility of this drug in ischemic stroke therapy, but more functional data of the human OCTs at the BBB is needed. 

## 6. Neuroprotective Properties of Memantine in Stroke

During ischemia, energy depletion occurs due to impaired delivery of oxygen and glucose to the CNS, a pathophysiological process that can increase cellular influx of cations to affected brain regions. Uncontrolled influx of calcium into neurons from brain extracellular fluid triggers release of glutamate, an excitatory neurotransmitter that is neurotoxic at high concentrations. Accumulation of glutamate in brain extracellular fluid is associated with neuronal cell death and development of an infarction [118]. One treatment strategy to enable neuroprotection in the setting of excitotoxicity is reduce excitatory effects of excess glutamate concentrations in ischemic brain tissue. This objective can be accomplished by targeting NMDA receptors with pharmacological inhibitors such as memantine. Memantine has a distinct advantage over other NMDA antagonists due to its fast on-off kinetics, low-to-moderate affinity for NMDA receptors, and its ability to attenuate excessive glutamate release without interfering with basal activation of NMDA receptors [119]. It has been proposed that memantine can also increase CNS expression of brain-derived neurotrophic factor (BDNF) and phosphorylated tropomyosin-related kinase B receptor (p-TrkBR), effects that can contribute to its neuroprotective properties [120]. Additionally, memantine reduces potential for neurocognitive impairments that can occur when activation of NMDA receptors is blocked, an effect that underscores its usefulness as a stroke therapeutic.

There are multiple preclinical studies that have described neuroprotective effects of memantine, as it is the prototypic transporter mediated neuroprotective drug. Studying the transporter mechanisms of memantine will undoubtably influence structure-based design for similar drugs with this desired mechanism of action. For example, in vitro studies in cultured chick retinal neurons showed that memantine (0–10 μM) conferred neuroprotection following injury induced by 30 min hypoxia/NaCN challenge. Neuroprotection was evaluated as an increase in cell viability, which occurred in a dose-dependent manner following memantine treatment [121,122]. In another study, 24 hours pretreatment with memantine decreased formation of striatal and striatocortical lesions in mice subjected to transient MCAO [119]. This study is particularly significant because memantine treatment was associated with both reduced lesion volume and improved neurobehavioral outcomes at 24 h post-MCAO. More recently, chronic treatment of memantine following photothrombotic stroke resulted in improved motor outcomes in mice as measured by the cylinder test and grid-walking test [120]. In assessing the impact of this study, it is critical to note that the photothrombotic stroke model produces early vasogenic edema that is not observed in human stroke. Therefore, photothrombotic stroke models are not considered to be appropriate for the measurement of neuroprotective efficacy of drugs. In contrast, data from MCAO studies provide encouraging evidence regarding the effectiveness of memantine as a stroke therapeutic.

## 7. Conclusions

Stroke is one of the most significant causes of death and long-term disability in the United States. At present, FDA-approved drug treatments for ischemic stroke are limited to r-tPA, which is not available to many patients due to its short therapeutic window (i.e., 4.5 h) and the risk of bleeding complications. Although recanalization in infarcted brain tissue is critical, these adverse events associated with r-tPA treatment are not trivial and can even promote neurological and vascular injury, thus exacerbating post-stroke neurological deficits. This indicates a need for stroke therapeutics that are both safe and effective as neuroprotectants; however, the clinical utility of such compounds is highly dependent upon efficient transport from systemic circulation into ischemic brain tissue. Indeed, the BBB possess several endogenous transporters that can be targeted to promote delivery of neuroprotective agents into the CNS. It is noteworthy that most studies on BBB transporters in stroke that have been published to date have primarily focused on transport of solutes involved in stroke pathogenesis such as ions and glucose. The next frontier of transporter biology will be to discern localization and functional properties of transport proteins at the BBB that can be targeted to optimize CNS drug delivery. Data from studies with statins and memantine indicate that OATPs/Oatps and OCTs/Octs represent transporter targets that can deliver drugs that are effective as stroke therapeutics to the brain. The fact that OATPs/Oatps are a known BBB uptake mechanism for statins is particularly striking since these drugs are well-known to be effective in improving neurological outcomes in stroke patients. This hypothesis has been reinforced by recent work with the opioid analgesic peptide biphalin, which is both a transport substrate for OATP1A2 [123] and capable of promoting neuronal recovery from experimental stroke [124]. Future development of neuroprotective treatment strategies for stroke with drugs will depend upon an improved understanding of discrete BBB transport mechanisms that will enable therapeutics to achieve effective brain concentrations. Information derived from BBB transport studies can be extended to inform discovery of new drugs developed specifically for ischemic stroke treatment. An examination of the chemical properties of statins and memantine that enable these drugs to be specifically transported by OATPs/Oatps or OCTs/Octs can inform structure-based drug design of such novel therapeutics. Overall, endogenous transporters at the brain microvascular endothelium must be studied in detail to discern the optimal time course and the most effective routes of administration for neuroprotective drugs. Furthermore, a consideration of biological variables (i.e., age, sex, comorbid conditions such as obesity, diabetes mellitus, atrial fibrillation, hypertension, etc.) that affect stroke outcomes should be incorporated into future experimentation to develop a better understanding of BBB transport mechanisms and, ultimately, improved strategies for treatment of ischemic stroke.

## Figures and Tables

**Table 1:** Strategies for treatment and prevention of ischemic stroke. Immediate (i.e., emergent) interventions for ischemic stroke include recombinant tissue plasminogen activator (r-tPA) infusion and endovascular thrombectomy (EVT). In addition to these approaches, preventative measures can be instituted for patients at high risk for stroke or to prevent recurrent stroke. Rehabilitative strategies are utilized to facilitate post-stroke recovery and to reduce the risk of recurrent ischemic stroke.

	Type	Goal
Immediate	**r-tPA (Alteplase)**	
	i.v. infusion Must be administered no more than 4.5 h after onset of stroke symptoms.	Fibrinolysis Restoration of perfusion of ischemic brain tissue
	**EVT**	
	Surgical approach where stent retriever is used to remove slot from occluded blood vessel. Must be conducted no more than 6 h after onset of stroke symptoms	Restore perfusion to ischemic grain tissue. EVT is commonly recommended for large vessel occlusion
Preventative	**Lifestyle changes**	
	Physical activity Smoking Cessation	20-30 min of aerobic exercise 5 days a week improves cardiovascular health. Reduces negative effects of tobacco on cardiovascular health
	**Anticoagulant/Antiplatelet Medications**	
	Warfarin (anticoagulant) Aspirin (antiplatelet)	Reduction in time to thrombolysis leads to reduced stroke incidence
	**Carotid Endarterectomy**	
	Surgical procedure involving removal of atherosclerotic plaque from carotid arteries.	Reduction in risk of new or recurrent ischemic stroke.
	**Cerebral Angioplasty or Stenting**	
	Surgical procedure involving either a balloon catheter (angioplasty) or insertion of a mesh steel brace (stent) into an occluded artery.	Increase cerebral reperfusion and reduce risk of recurrent ischemic stroke.
Rehabilitative	**Pharmacotherapy**	
	Statins Antihypertensive Drugs ACE inhibitors	Improve neurocognitive recovery Reduce risk of recurrent stroke
	**Supportive Care**	
	Speech therapy Occupational therapy Physical therapy	Improve motor and cognitive functionality

**Table 2:** A list of representative substrates for the solute carrier family 21 (SLCO) transporter OATP1A2. Endogenous substrates are on the right panel. Frequently prescribed drugs that are substrates for OATP1A2 are listed in the middle and left panel. Drugs are clustered by therapeutic class and this list in not exhaustive. Endogenous transport substrates of OATP1A2 include bile acids and linear and cyclic peptides, steroid conjugates, the thyroid hormones T4, T3 and rT3, prostaglandin E2. Pharmacologic and other exogenous transporter substrates for OATP1A2 include antibiotics, synthetic prostaglandins, HIV antivirals, 3-hydroxy-3-methylglutaryl coenzyme A (HMG-CoA) reductase inhibitors (statins), natural product cardiac glycosides, antihistamines, beta adrenergic blocking agents (beta blockers), chemotherapeutics, the synthetic opioid receptor agonists [D-Pen2,D-Pen5]enkephalin (DPDPE) and deltorphin II, certain magnetic resonance imaging contrast agents, and the dye sulfobromophthalein (SBP).

Endogenous Substrates	Drug Substrates	
**Steroid Hormones:**	**Antibiotics:**	**Beta-blockers:**
DHEA-S Estradiol-17β-glucuronide Estrone-3-sulfate	Erythromycin Ciprofloxacin Gatifloxacin Rifamycin Levofloxacin/fluoroquinolones Tebipenem Pivoxil	Acebutolol Atenolol Celiprolol Labetalol Nadolol Sotalol Talinolol
**Thyroid Hormones:**	**Prostaglandins:**	**Chemotherapeutics:**
Reverse triiodothyronine (rT_3_) Thyroxine (T_4_) Triiodothyronine (T_3_)	Unaprostone	Methotrexate Imatinib Atrasentan
**Prostaglandins:**	**HIV antivirals:**	**Dyes:**
Prostaglandin E_2_	Darunavir Lopinavir Saquinavir	Sulfobromophthalein (SBP)
**Bile Acids:**	**Statins:**	**Paralytics:**
Bilirubin Cholic acid Glycocholate Taurocholate Taurochenodeoxycholate (TCDC) Tauroursodeoxycholate (TUDC)	Rosuvastatin Atorvostatin Pitavastatin	Rocuronium
**Other:**	**Cardiac Glycosides:**	**Opiates:**
linear and cyclic peptides	Ouabain	Deltophorin II DPDPE (synthetic)
	**Antihistamines:**	
	Fexofenadine	
